# Implementation of Wi-Fi Signal Sampling on an Android Smartphone for Indoor Positioning Systems

**DOI:** 10.3390/s18010003

**Published:** 2017-12-21

**Authors:** Hung-Huan Liu, Chun Liu

**Affiliations:** Department of Electronic Engineering, Chung Yuan Christian University, Taoyuan 32023, Taiwan; jim83803@gmail.com

**Keywords:** indoor positioning system, quick radio fingerprint collection, neighboring vertices averaging, Wi-Fi

## Abstract

Collecting and maintaining radio fingerprint for wireless indoor positioning systems involves considerable time and labor. We have proposed the quick radio fingerprint collection (QRFC) algorithm which employed the built-in accelerometer of Android smartphones to implement step detection in order to assist in collecting radio fingerprints. In the present study, we divided the algorithm into moving sampling (MS) and stepped MS (SMS), and describe the implementation of both algorithms and their comparison. Technical details and common errors concerning the use of Android smartphones to collect Wi-Fi radio beacons were surveyed and discussed. The results of signal sampling experiments performed in a hallway measuring 54 m in length showed that in terms of the amount of time required to complete collection of access point (AP) signals, static sampling (SS; a traditional procedure for collecting Wi-Fi signals) took at least 2 h, whereas MS and SMS took approximately 150 and 300 s, respectively. Notably, AP signals obtained through MS and SMS were comparable to those obtained through SS in terms of the distribution of received signal strength indicator (RSSI) and positioning accuracy. Therefore, MS and SMS are recommended instead of SS as signal sampling procedures for indoor positioning algorithms.

## 1. Introduction

In the 2000s, the growth of wireless sensor network technologies led to several new applications that provide location-based services (LBSs) to better serve users based on user context and location. Among these applications, Wi-Fi-based indoor positioning and navigation services have attracted considerable attention because their system installation costs are affordable and they offer acceptable positioning accuracy for some LBSs [1,2]. Wi-Fi is used in many territories in such a manner that access points (APs) are very cheap and easy to obtain. Wi-Fi APs have been deployed in many buildings, campuses, and underground facilities. Meanwhile, the computational capacities of Wi-Fi-embedded mobile devices such as iPhone and Android are rapidly gaining ground, and device costs continue to drop alongside the rapid development of personal mobile applications and services. 

Wi-Fi-based positioning methods are currently grouped into three main categories based on the positioning principles of proximity, trilateration, and scene analysis. The proximity positioning method regards the position of the AP with the strongest received signal strength indicator (RSSI) as the user’s location. Although such an algorithm is relatively simple and fast, its accuracy is low compared with other methods [3]. Generally, positioning error is positively correlated with the density of AP provisioning, and the locations of APs in a building must be provided in advance. The proximity method has received extensive attention among researchers of wireless beacon-based indoor positioning systems, such as Bluetooth Low Energy (BLE) beacons.

The trilateration positioning method uses three or more AP locations to calculate distances based on differences in the time or strength of received signals. The calculated distances are then used to estimate a user’s location. The current Wi-Fi system can operate only with the RSSI method, which uses a wireless transmission attenuation model to calculate the distance from the estimation point to the AP. The multipath effect generated from wireless signals in an indoor environment makes it considerably more difficult for the trilateration method to achieve high-accuracy positioning, resulting in failure to obtain acceptable levels of accuracy and precision. Some studies have dedicated their efforts to improving positioning accuracy based on trilateration [4,5].

The scene analysis positioning method is generally divided into two phases. In the first phase (the “offline phase”), signals are sampled offline. Several positions in a building (called “sample points”) are determined in advance. The RSSI of each AP at these sample points and its basic service set identification (BSSID) is sampled and recorded in a database (called “radio fingerprint”) along with each sample point’s coordinates. In the second phase (called the “position estimation phase”), a user uses a smartphone to collect each AP’s RSSI and BSSID at a specific located point of interest. These measurements are compared with the measurements stored in the radio fingerprint to determine the user’s position. The nearest neighbor in signal space algorithm was proposed in [6] as a means of computing the signal space distance between the observed and recorded measurements. The nearest neighboring sample point in signal space is regarded as the user’s position. The advantage of this approach is its ability to reduce multipath problems [7]; however, when using this method, the intensity of the sample points directly affects positioning accuracy.

A crucial problem of scene analysis positioning is the high cost of the offline radio fingerprint building process, which is a time-consuming task. In the traditional approach, creating a coordinate system for the building in question, marking the location of every sample point with a measuring stick or tape, and then collecting the AP information at each sample point one by one are all necessary measures. Generally, at each sample point, several tens of samples are performed and the average RSSI is used as the value recorded in the fingerprint database. This procedure is named as the static sampling (SS) method in this study. Sometimes, to prevent the human body from absorbing the Wi-Fi signal, the data collection procedure requires the signal to be sampled from four directions, after which the average is used as the final record [6]. In a building, increasing the number of sample points directly increases the positioning accuracy of the scene analysis method; however, this measure also increases the cost of creating the fingerprint database.

A common strategy for calculating Wi-Fi signal strength is the use of probability calculation to construct likelihood models of signal strength measurements. Ferris et al. proposed Wi-Fi simultaneous localization and mapping (Wi-Fi-SLAM) [8], which moves along predetermined paths on a map to collect Wi-Fi signals, pinpoint the location of ground truth on a handheld device, and define a kernel function between the signals so that Gaussian process (GP) latent variable models can be used to compute the distribution function for the signals. However, the authors did not state how long they took collecting Wi-Fi signals. Additionally, because information regarding the collection of Wi-Fi signals by using cell phones was not completely disclosed in 2007 (the relevant details are provided in Section 2), all six positioning experiments in that study yielded a mean error of 3.97 ± 0.59 m. Because the Wi-Fi signals are collected while in motion, these signal sampling procedures can be referred to as moving sampling (MS).

Other SLAM techniques have been applied in previous studies [9,10] with median errors ranging from 6.7 to 8 m. However, because of their limited precision, these techniques are unsuitable for indoor positioning. To improve the positioning precision of Wi-Fi-SLAM, Yiu and Yang [11] developed a method for Wi-Fi signal reconstruction that involves incorporating the firefly algorithm into a GP algorithm. The researchers used an SS-based method to collect Wi-Fi signals from a few sampling sites to reconstruct the signal strength distribution of an entire field. In their experiment, the median error was approximately 3 m.

In MS, the sample point is different for each sampling and only one Wi-Fi sample is collected from each individual sample point. Compared with SS, which can derive an average from multiple samples collected from a single position, MS is therefore more likely to have a higher sampling error. Thus, we proposed a quick radio fingerprint collection (QRFC) algorithm [12] that uses AP RSSI shaping rather than the GP algorithm to calibrate collected Wi-Fi signals. Despite having a rigorous mathematical basis, the GP algorithm requires a kernel function to be provided in order to describe the signal correlation between two sample points. In practice, providing a blanket kernel function for all indoor environments is difficult because of the sheer complexity of such environments. On the other hand, the strength of signals received by Android phones is expressed in dBm (see Section 2 for further details); therefore, algorithms based on probability models are subject to serious quantization errors at the beginning of the signal sampling process. Although AP RSSI shaping is a heuristic algorithm, it features low calculation load and acceptable positioning accuracy. Moreover, the QRFC and SS methods have been experimentally proven to be comparable in terms of positioning accuracy for Wi-Fi signal collection, and both can be successfully applied to navigation and indoor positioning [12].

In this paper, the implementation of MS and stepped MS (SMS) are illustrated. Additionally, the causes and effects of common problems inherent in Wi-Fi signal sampling and proposed solutions are discussed. The findings of this study are expected to aid researchers studying the application of Android smartphones to indoor positioning and provide them with an understanding of how to collect appropriate signals through simple methods, reduce errors during programming and the use of research instruments, and improve the accuracy of their experimental results.

The remainder of this paper is organized as follows. In Section 2, problems during Wi-Fi signal sampling and the proposed framework for Wi-Fi positioning are introduced. In Section 3, the application of SS, MS, and SMS and the respective analyses of Wi-Fi signals collected using these three methods are described. Section 4 presents the experimental results and a related discussion. Conclusions are offered in Section 5.

## 2. Wi-Fi Signal Sampling: Implementation and Environmental Impact

This section presents our survey of the scanning and reporting procedures for Wi-Fi signals in Android-based smartphones, and discusses the problems that these procedure cause in the signal sampling of indoor positioning systems. Section 2.4 describes the step detection algorithm employed in this study to aid the computation of locations for Wi-Fi signal collection.

### 2.1. Wi-Fi Signal Scanning by Android Smartphones

**Figure 1:**
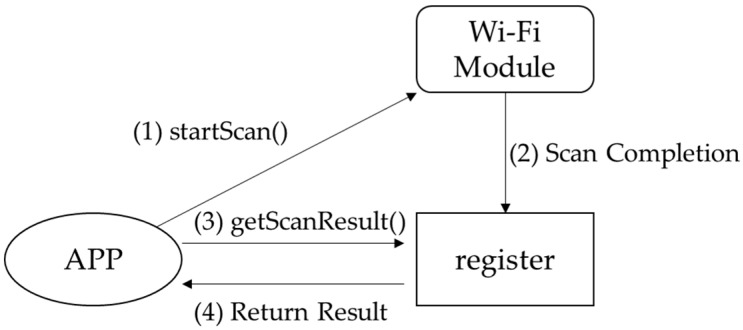
Simplified procedure for Wi-Fi signal scanning using Android.

Figure 1 shows a framework for the scanning of Wi-Fi signals by Android smartphones. For brevity, the figure illustrates only how the system processes the Wi-Fi signal and does not show how it processes the intermediary signal. The procedure for the system’s scanning of the Wi-Fi signal is described as follows:

(1) The app sends a startScan() command to the Wi-Fi module to scan for nearby AP signals.
(2) When the Wi-Fi module has finished scanning the signals, it stores its scanning results in the cache. Subsequently, a notification reading “Scan Completion” is sent to the operating system (OS).
(3) The OS notifies the app of the completed scan. The app then sends a getScanResults() command to request the scanning results stored in the cache.

In this procedure, the app may read incorrect scanned data on two occasions. First, when the app sends the startScan() command, the Wi-Fi module takes a few seconds to finish scanning the AP signals (approximately 3 s on an HTC One M8 and 4 s on a Samsung S5, according to the results of our experiment) and ignores other startScan() requests until AP signal scanning has been completed. Moreover, because the getScanResults() command is independent of startScan(), using the getScanResults() command to read data stored in the cache immediately after the startScan() command is issued results in accessing only data stored in the cache from the previous scan. That is to say, whenever the app sends the getScanResults() command, it reads the data in the cache. Second, the Wi-Fi module updates some data in the cache while keeping some intact; for example, if the cache contains three AP signals—AP1, AP2, and AP3—and only AP1 and AP2 were acquired in the most recent scan, the signal data of AP1 and AP2 only are updated. The details of the cache updating algorithm are unknown. When using an HTC One M8, Samsung S5, or Nokia 6, the lifetime of the caching data is around 10 s, and sometimes up to 30 s.

Because of the aforementioned conditions, when the getScanResults() command is used to access Wi-Fi signal scanning results, signals collected from incorrect locations at incorrect times are likely to be acquired, and thus errors may occur in data analysis. Furthermore, because the Wi-Fi module takes 3–4 s to process the startScan() command to acquire new scan results, if a study used methods in which claim to perform sampling per second and no subsequent filtering was performed on the obtained AP data, the AP data would contain repeated data, resulting in erroneous statistical analyses.

This limitation could lead to more severe consequences for phones using MS. Specifically, Wi-Fi signals collected by a smartphone through MS may have been signals pertaining to the position 3–4 s ago, and this would undermine the accuracy of the final position. However, such limitations were resolved in September 2012, when the Android OS was updated to version 4.2 (Jelly Bean MR1) with an application programming interface level of 17. In this version, the timestamp field was added as a new feature for logging the AP signal data obtained through the getScanResults() command on a microsecond timescale, which indicates when this result was last seen. This feature enables users to determine whether the obtained data are up to date.

### 2.2. Impact of Sampling Sites

**Figure 2:**
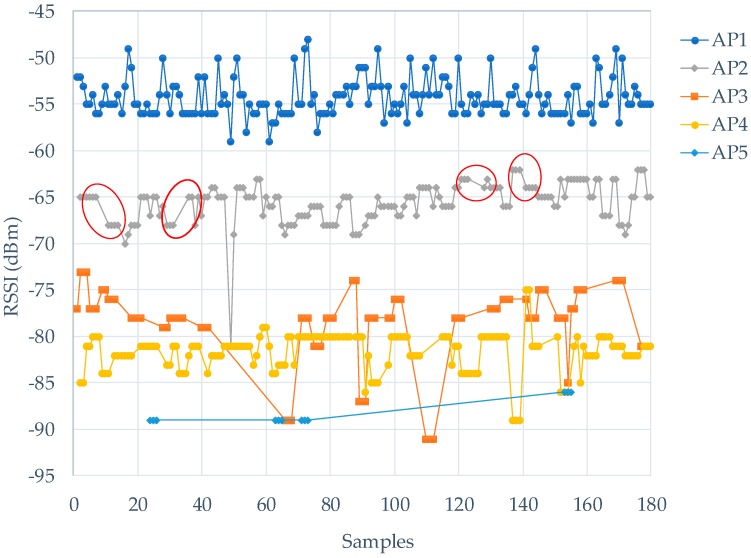
Detected received signal strength indicator (RSSI) sequences at a specific sampling point.

The convenience of Wi-Fi technology has led to the establishment of a growing number of APs, and consequently a highly crowded AP environment. Moreover, since Wi-Fi technology has only at most three independent channels (for the 2.4-GHz band), APs interfere with one another to vie for the channels. From the viewpoint of Wi-Fi stations, this means some AP signals appear at certain times and disappear at others. An experiment shown in Figure 2 involves sampling at a given sample point for 180 samples. In Figure 2, AP signals use Channel 1 (2.412-GHz band), and the beacon signal of AP1 is one of the strongest RSSIs in this place, and all samples include the fresh signal from AP1. AP2 is not detected by the smartphone in a few samples; we indicated it with a red circle. The phenomenon is more obvious when AP with lower RSSI like AP3, 4, and 5 in Figure 2. Therefore, smartphones likely stochastically detect AP signals. Positioning algorithms used in smartphones should prevent this stochasticity.

### 2.3. Smartphone Wi-Fi Modules are Prone to Errors in Wi-Fi Signal Strength Measurements

A Wi-Fi module for a smartphone is designed to receive and send signals and must be energy efficient. However, no rigorous standards have been established for the measurement of Wi-Fi signal strength by smartphones. Consequently, signal strength measurement results reported by smartphones are prone to errors. Table 1 shows the mean RSSI values and standard deviations for three smartphones used to collect Wi-Fi signals for 60 s from one position at the same time. Four APs are selected according to different RSSI levels. The experiment was heavily affected by the environmental influence of the experimental site; therefore, the numerical results varied markedly between measurements. These results are intended to highlight the different smartphone brands and Wi-Fi chipsets will report different RSSI values in smartphone-based Wi-Fi signal measurements rather than assess device performance.

Additionally, in Android smartphones, the Wi-Fi signal strength measurements are quantized with the step of 1 dBm. Thus, the exponential decay equation for Wi-Fi signals can be used to derive a relational formula for the distance between 1 dBm signal gap in a given geometric space.

According to the formula of exponential decay equation for wireless signals:

(1) $$P(d)=P(d_{0})-10\log{\left(\frac{d}{d_{0}}\right)}^{\alpha }-\mathrm{OAF}$$

where *P*(*d*) represents the signal strength at distance *d* from the position of the transmitter; *P*(*d*_0_) represents the signal strength at distance *d*_0_ from the position of the transmitter, which is the distance (called the “reference distance”) at which the intensity of the signal’s strength can be measured and recorded in advance; α is the channel attenuation coefficient; and *OAF* is the obstacles attenuation factor, which contains the collective influences from the multi-path effects caused by internal barriers such as walls and partitions. Assume that there are two points, their distance from the antenna is *d*_1_ and *d*_2_ respectively, and with 1 dBm RSSI strength gap. Thus, |*P*(*d*_1_) − *P*(*d*_2_)| = 1 dBm and the relation between *d*_1_ and *d*_2_ is:

(2) $$|\log\left(d_{2}\right)-\log\left(d_{1}\right)|=\frac{1}{10\times \alpha }.$$

Generally, whereas α ranges between 2.7 and 3.5, the *d*_2_ to *d*_1_ distance measures between 0.2 and –1.5 m, which changes according to the distance to the antenna. Positioning algorithms should address such measurement and quantization errors.

### 2.4. Step Detection

**Figure 3:**
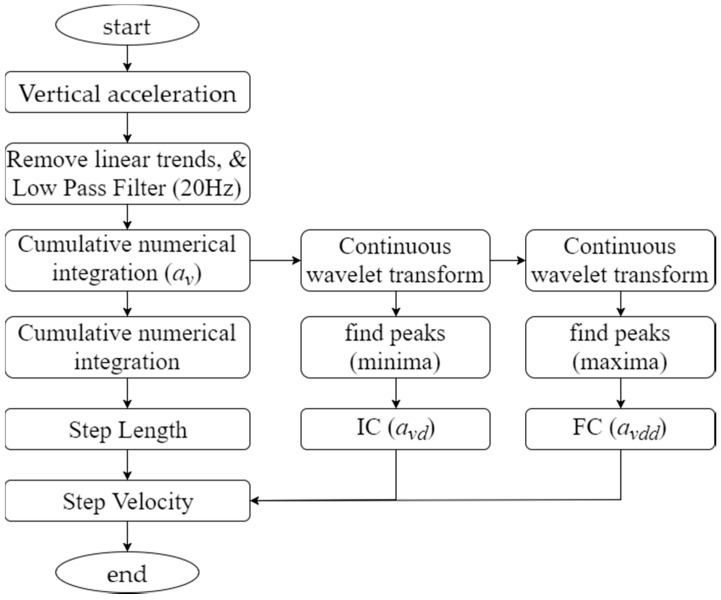
Procedure for processing accelerometer signals for gait detection.

Android smartphones have been retrofitted with sensors such as accelerometers and gyroscopes, and such sensors have been used in research to improve the precision of indoor positioning [13]. However, this study used the step detection system to aid Wi-Fi signal collection and established a database of radio fingerprints rather than applied the system to positioning algorithms. Kitkat and later versions of the Android OS included a step sensor that enables step detection and counts steps, but this sensor takes approximately 2 s to report events, and thus is not helpful for Wi-Fi signal collection. In our previous study [12], we used the simple step detection algorithm proposed by Libby [14]. This algorithm enables smartphones with limited computation capacity to perform instant calculations. In that study, the signal sampling algorithm for the indoor positioning system performed batch calculations after sampling signals, thereby eliminating the need for instant calculations. Therefore, we had used the methods proposed by Din et al. [15] and Moe-Nilssen [16] to conduct a step detection procedure with accuracy higher than that of Libby’s proposed algorithm. In Moe-Nilssen’s research, accelerometer signals were transformed into a horizontal–vertical coordinate system. Din et al. extracted vertical components and used a fourth-order Butterworth low pass filter [17] at a cutoff frequency of 20 Hz and wavelet transform to identify initial contact (IC) and final contact (FC) within a gait cycle. Moreover, when the distance from an accelerometer to the ground is known, an inverted pendulum model [18] can be employed to estimate step length and velocity. Figure 3 depicts the procedure for processing accelerometer signals.

## 3. Wi-Fi Signal Sampling

The Wi-Fi signal sampling methods used in the present study were divided into three types: SS, MS, and SMS. SS is the traditional method for capturing Wi-Fi signals, which requires constructing a coordinate system for the indoor environment, marking target sampling points, and collecting signals at each point in order. This sampling process is performed based on a fixed timeline and at a predetermined rate (e.g., scan once per second for one minute). As described in Section 2.1, an Android smartphone takes 3–4 s to complete a valid Wi-Fi signal scan. Many studies have presented results of sampling per second and provided no explanation for the processing of valid and invalid signals. Moreover, if getScanResult() is sent once per second, the user acquires considerable overlapping data, which renders the statistical analysis of sampled signals erroneous. In the present study, SS was performed with startScan() once per second; however, the smartphones used in the sampling procedure acquired signal data at intervals of 3–4 s depending on their design. For each minute of SS, 15–20 valid signals were collected.

**Figure 4:**
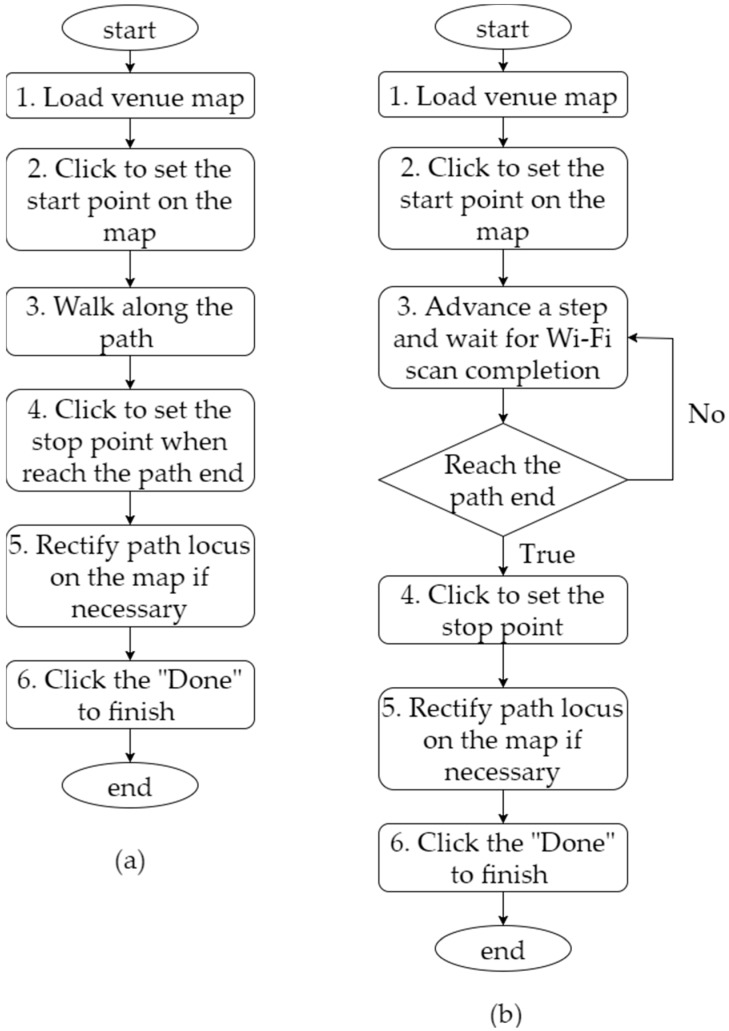
Wi-Fi signal procedures: (**a**) moving sampling (MS) and (**b**) stepped MS (SMS).

In MS, the route inside a building is predetermined, and the measuring user moves along this route at a speed slightly slower than the general walking speed. When the user is moving, the Android smartphone sends the startScan() command every second and collects Wi-Fi signals according to the procedure described in Section 2.1. Figure 4a illustrates six steps in MS. First, an app is initiated to load the map of the sampling site. The map is shown on the screen of the phone. Second, the user proceeds to the starting point of the route established for the target sampling location, clicks to set the point on the map, and presses “Start” on the screen to initiate the phone’s sending of a scanning signal (startScan()) every second. Third, the user remains at the starting point for about 5 s (so that the phone can complete its first scan and acquire the first record of signal data) and walks slowly to the end of the route. Fourth, upon reaching the end of the route, the user remains there also for about 5 s (for the phone to complete its scan) and clicks to set the end point on the map and terminate the entire scanning process. Fifth, the app charts and presents the path from the starting point to the end point of the route. The path can be manually modified if necessary. Finally, the user presses “Done” on the screen to finish scanning the route.

Similar to MS, SMS involves predetermining the sampling routes inside a building. However, what distinguishes these two methods is that in SMS, for every step taken during sampling, the user has to stop and send the startScan() command manually and should not take the next step until the phone has acquired the signal data and responded with the message “Scan Completed.” It takes 3–5 s to acquire data in each step. As Step 3 in Figure 4b shows, when advancing a step forward, the user waits for the phone to send the startScan() command and acquire the latest Wi-Fi scan data. Subsequently, the app on the phone delivers the “Scan Completed” message, and the user proceeds. SMS is sometimes viewed as a variant of SS where sampling occurs once for each step.

In this study, the sampling route length was set to 54 m. SS was performed once for 1 min in each meter of the route. Coupled with the transfer of requisite equipment, the sampling procedure took approximately 120 min. Conducting MS and SMS on the route took approximately 150 and 300 s, respectively. Table 2 compares the amounts of time taken to complete MS, SMS, and SS in the 54-m sampling route.

After collection, Wi-Fi AP signals were preprocessed to filter out false signals, including (1) service set identifiers with keywords such as “iPhone” and “Android,” which might have come from smartphones serving as temporary Wi-Fi hotspots by enabling their 3G- or 4G-network bridges; and (2) signal data with expired timestamps.

### 3.1. Signal Mapping

When the MS method is used, the timestamp of each IC recorded by the accelerometer is calculated and collected. Because the starting and end points of all sampling routes are already known, if the user’s stride length is fixed, the temporal positions of IC events for each route can be established. Subsequently, the exact location corresponding to the each recorded Wi-Fi RSSI data can be inferred through the accompanying timestamp. Assume that the respective coordinates of the starting and end points are ***X*_0_** = (*x*_0_, *y*_0_) and ***X_n_*** = (*x_n_*, *y_n_*), the number of steps taken is *n*, the step length is *L* and a constant. If the route is straight, ***L*** = (***X_n_*** − ***X*_0_**)/*n*. Under these circumstances, the coordinates (***X_i_*** where *i* = 0, 1, …, *n*) of each IC on the map and the occurring time (*t_ic_*_,*i*_ where *i* = 0, 1, 2, …, *n*) can be identified.

If the timestamp (*t_w_*) for a given Wi-Fi RSSI record falls between two instances of IC (*t_ic_*_,*j*_ ≤ *t_w_* ≤ *t_ic_*_,*j+*1_, 0 ≤ *j* ≤ *n*), this record is assumed to had been established between ***X_j_*** and ***X_j_*_+1_** with the ratio of (*t_w_* − *t_ic,j_*)/(*t_ic,j_*_+1_ − *t_w_*) approximate to that of (*x_w_* − *x_j_*)/(*x_j_*_+1_ − *x_w_*), where the *x_w_* is the estimated position of the sampling point.

**Figure 5:**
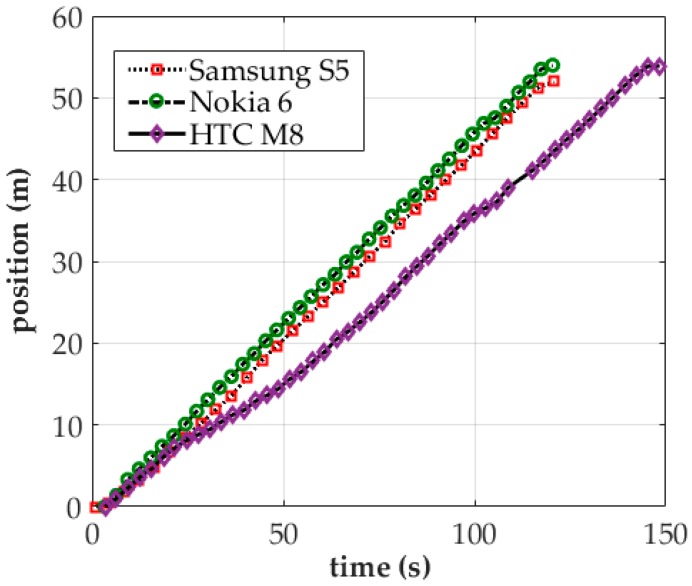
Time–position distribution of MS on a Samsung S5, Nokia 6, and HTC M8.

Figure 5 presents the time–position distribution of MS as operated by a Samsung S5, Nokia 6, and HTC M8 on a given route. The x-axis of the figure represents the scan completion reporting time and the y-axis denotes the reporting position. The sampling procedure lasted longer on the HTC M8 (approximately 150 s) than on the Nokia 6 and Samsung S5 (both 120 s). The number of valid scans completed was 49 on the HTC M8, 40 on the Nokia 6, and 31 on the Samsung S5. On average, the Nokia 6 and HTC M8 reported scanning results every 3 s, whereas the Samsung S5 did so every 4 s.

**Figure 6:**
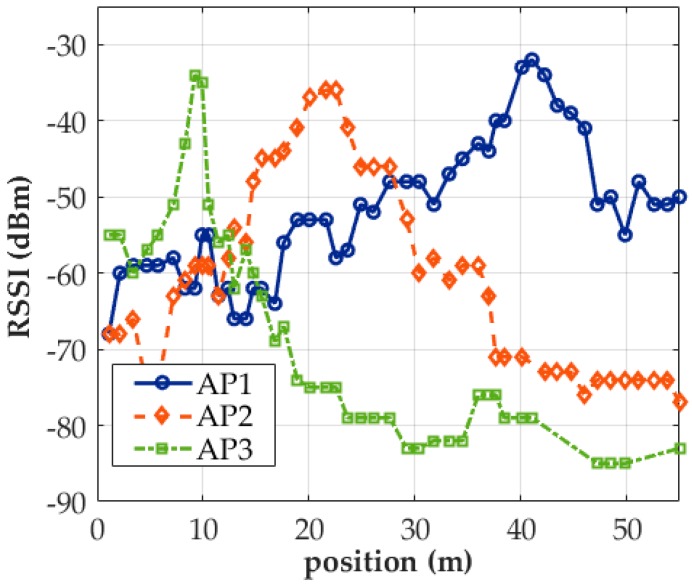
Distributions of AP signals collected at the 2.4-GHz band through MS (markers on the curves indicate where valid data were collected).

Figure 6 depicts the RSSI distribution of three APs obtained using MS by HTC M8. AP1 and AP2 were used to establish a Wi-Fi service on campus. The APs sent strong signals over a long distance, and thus their RSSI distributions declined steadily. AP3 was an AP router installed in a lab. The AP had lower output power and its RSSI distribution declined rapidly in comparison with those of the other two APs. Not all scan reports contained complete signals. Of the 49 scan reports from this experiment, 48, 47, and 41 contained the AP1, AP2, and AP3 signals, respectively. Additionally, the AP3 signal was not detected at many sampling positions between 40 and 50 m.

Certain details regarding MS implementation should be noted. First, when the MS app is run on the multitasking Android OS, other apps may be running simultaneously in the background. The user should disable as many of these background-running apps as possible to enable the MS app to operate steadier. Second, other apps should be prevented from using the Wi-Fi module lest they interfere with the MS app. This can be achieved by setting WifiManager.setWifiEnabled() to be “false” (disabling the Wi-Fi interface) and WifiManager.createWifiLock() to be “WifiManager.WIFI_MODE_SCAN_ONLY” (confining the Wi-Fi module to Wi-Fi scanning). Performing MS without these settings in place may result in signal data that are unsuitable for use as samples for radio fingerprints. 

**Figure 7:**
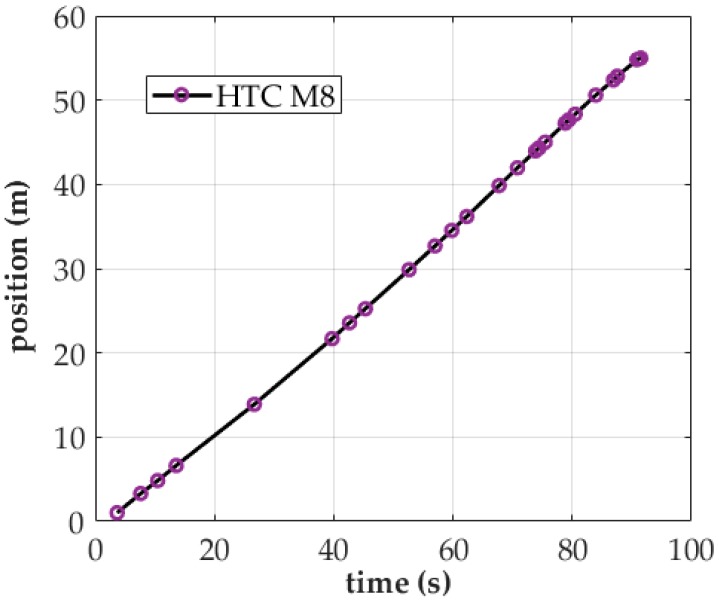
Time–position distribution of MS on the HTC M8 (Wi-Fi interface enabled).

Figure 7 shows the MS results obtained using the HTC M8 and without setting the aforementioned two parameters. In this experiment, it took only 96 s to collect signals along the entire 54-m sampling route. Although the sampling procedure was completed in a relatively short time comparing with Figure 5, the time factor did not influence the sampling results. As Figure 7 indicates, only two scan reports are found at the sampling time of 15–40 s, likely because other apps (e.g., updates regarding activity in a Facebook or Gmail account) were using the phone’s Wi-Fi interface during the same period. By contrast, the two instances in which high activity in scan reports occurred at a sampling time of 70–80 s may have been scan reports cached when the OS was occupied and could be sent only later. Based on the results, signal data obtained through MS should not be used to create radio fingerprints unless the parameters are properly set.

**Figure 8:**
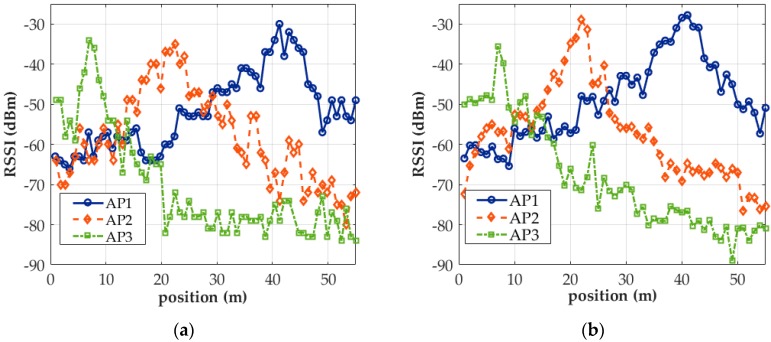
Distribution of AP signals collected: (**a**) through SMS; (**b**) through SS. (at the 2.4-GHz band).

During SMS, one valid Wi-Fi signal was acquired from the location where each IC occurred. SS lasted for 1 min at each 1-m gap between sampling positions and yielded the mean of the RSSI values in this study. A tripod was used to aid SS and reduce human interference in signal collection. SS and SMS both eliminate the need to reconfigure the initial status of a smartphone or limit the number of apps running on it. Both have high error tolerance from operational mistakes but take longer time to complete scanning. Figure 8a,b presents the respective distributions of signals collected from APs 1, 2, and 3 through SMS and SS by HTC M8. Notably, the RSSI values of AP signals acquired through SMS changed in a highly similar manner to those of AP signals acquired through SS. Even when 20 SS-collected signals were averaged, the RSSI distribution showed a zig-zag pattern; in other words, it is difficult to ascertain from Figure 8a,b alone as to which panel is associated with which sampling method.

### 3.2. Signal Shaping

According to the formula for wireless signal attenuation, signal strength is inversely proportional to the α-th power of distance (the α of an indoor environment is 2.7–3.5). When a strong signal is detected, its peripheral signals are comparable in strength unless a serious interference occurs. Based on this rationale, this study proposed a signal shaping algorithm called neighboring vertices averaging (NVA) [12] to modify collected signals for subsequent position estimation algorithms. In the NVA algorithm, a line segment connecting two vertices of the raw RSSI is drawn. If a vertex is located between the two end vertices and beneath the line segment, the point on the line segment is selected as the RSSI of the shaped version, otherwise the raw RSSI is selected as the RSSI of the shaped version. Whole pairs of vertices in the raw RSSI are checked to find the envelope of the raw RSSI. Because the signal attenuation of wireless signals exhibits exponential decay, the NVA algorithm can approximate the exponential decay only in a small region. Consequently, a window size is used to limit the maximum connectable vertex distance in NVA. In our experiment, the window size was set to 5 m. The details of the algorithm (Algorithm 1) are shown as follows:
**Algorithm 1:**Preliminary:1.Set the sequence of raw RSSI to follow the sampling sequence and is described as *S* = {(***X***_0_, *r*_0_), (***X***_1_, *r*_1_), …, (***X****n*, *r_n_*)}, where ***X*** is a coordinate position and *r* is an RSSI value. If *r_p_* denotes the highest RSSI value, 0 ≤ *p* ≤ *n*. Thus, the right and left halves respectively form two different sets denoted respectively by *S_R_* and *S_L_*, defined as follows: 2.*S_R_* = {(***X****_p_*, *r_p_*), (***X****_p+_*_1_, *r_p_*_+1_), …, (***X****_n_*, *r_n_*)} 3.*S_L_* = {(***X****_p_*, *r_p_*), (***X****_j_*_−1_, *r_p_*_−1_), …, (***X***_0_, *r*_0_)} 4.Set the windows size to *w*.*S_L_*:1.All *r* values are copied once and named *sr.*2.for each *i* = *p* to *n*−13. for each *j* = *i* + 2 to *k*, where $\underset{k}{\max}\{X_{k}-X_{i}\leq w\}$4.  Draw a straight line connecting *r_i_* and *r_j_*.5.  If *sr_a_* is lower than this line, *sr_a_* is updated as a value for the line at ***X****_a_*, where *i* < *a* < *j*.6. end *j*7.end *i*8.Correct the margin as follows: 9.if (*r_n_* ≥ *sr_n_*_−1_),10. do nothing11.else 12. Extend the line connecting *sr_n−_*_2_ and *sr_n−_*_1_ to ***X****_n_*. If *sr_n_* is lower than the line, 13. *sr_n_* is updated as a value for the line at ***X****_n_*.*S_R_*:14.Repeat the steps performed for *S_L_* but in the opposite direction.Output: 15.Save *sr* as the modified RSSI distribution.

## 4. Results and Discussion

**Figure 9:**
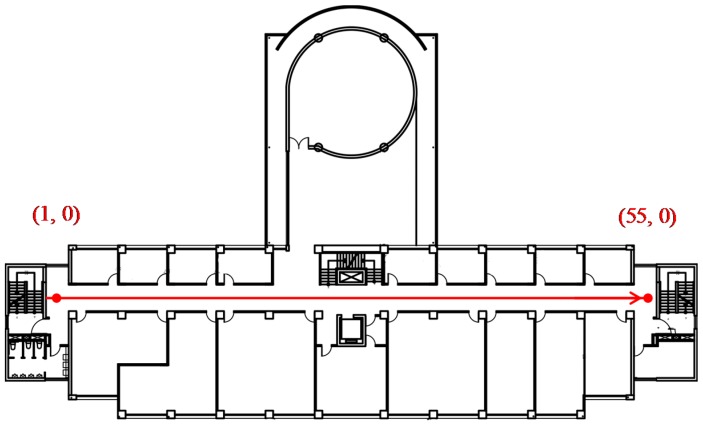
Floor plan of the experimental site.

The experiment was conducted on the fifth floor of the Electrical Engineering and Computer Science Building of Chung Yuan Christian University in Taoyuan City in northern Taiwan. Figure 9 shows the floor plan of the experimental site. The building has eight floors. Each floor has staircases on the left- and right-hand sides and another stairway and elevator in the middle. The fifth floor has an open space for students to engage in recreational and academic activities. We collected AP signals on a path from (1, 0) to (55, 0) along the hallway of this floor. In this building, over 40 AP signals were captured in each scan, totaling more than 600 APs, thereby indicating that AP distribution is dense.

**Figure 10:**
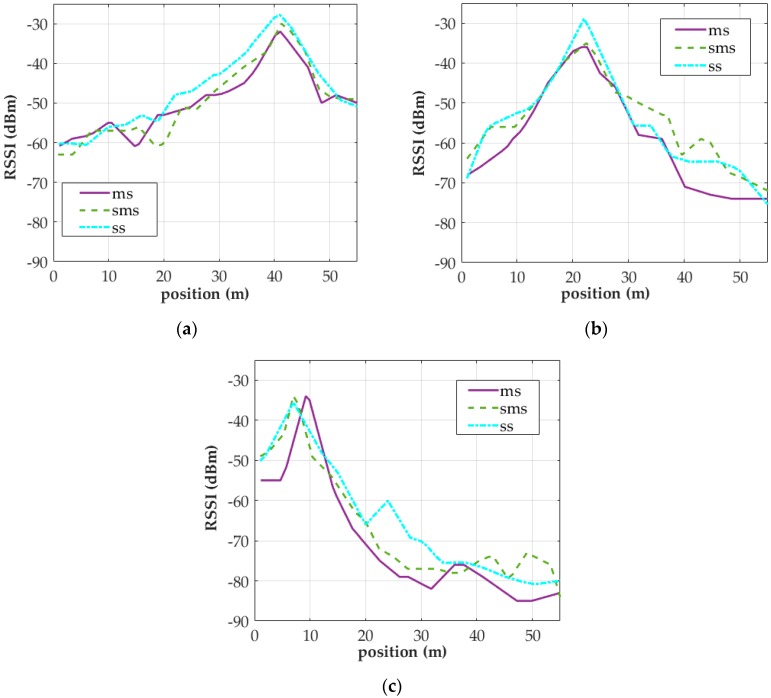
RSSI distributions of signals collected from various APs (the distributions were derived through the neighboring vertices averaging (NVA) algorithm): (**a**) AP1; (**b**) AP2; (**c**) AP3.

Three of the strongest AP signals are chosen to explain the results. Stronger signals played a crucial role in the subsequent positioning algorithms, whereas weaker signals were eliminated from the algorithms. The NVA algorithm yielded the RSSI distributions for APs 1, 2, and 3 (Figure 10). The RSSI distributions for APs 1 and 2 obtained using MS, SMS, and SS were similar. However, higher RSSI was measured by SS because a tripod was used during the sampling process to position a smartphone approximately 120 cm above ground to prevent the researcher’s presence from interfering with the collected signals. By contrast, signals collected using MS and SMS were influenced by the researcher’s presence because the researcher held the smartphone during the sampling processes. Nonetheless, the strength of these signals more accurately reflected real conditions because the smartphones were held on hand by the user during movement and positioning.

The fingerprint database of an indoor positioning system requires regular updates to integrate newly acquired and existing samples and converge them into a stable version. Therefore, when the database is updated multiple times, the RSSI distributions of signals from different APs become increasingly similar.

In Figure 10c, the peak RSSI distribution for the AP3 signal acquired using MS was displaced to the right by approximately 2 m from the positions of the AP3 signals obtained using SMS and MS, and the width of main lobe is narrower than the other two. This deviation was due to a few number of steps were not counted in the step detection algorithm results during sampling. In addition, the accuracy of the algorithm affects the RSSI distributions of MS-acquired signals. For example, if the algorithm fails to precisely estimate the signal sampling points interpolated between two instances of IC, the resulting RSSI distribution displaces to the left or right, narrower or wider. Thus, the accuracy of the step detection algorithm employed in this study was also tested.

During the test, three MS procedures were implemented. In the first procedure, the tester held the smartphone approximately 30 cm away from his or her chest or the typical viewing position. In the second procedure, the tester used a FlipBelt [19] to attach the smartphone to the fifth lumbar vertebra and used Bluetooth to operate when to begin and terminate signal scanning. In the third procedure, the smartphone held by hand was mounted onto on a smartphone stabilizer (Osmo Mobile [20]) to prevent vibrations during sampling. According the experimental results presented in Table 3, the results obtained using the smartphone stabilizer showed the lowest accuracy rate, whereas a 100% accuracy rate was achieved for all four tests conducted by attaching the phone to the fifth lumbar vertebra. However, because the human body absorbs large amounts of signals, although attaching the phone to the back yielded more accurate step detection, it also resulted in collecting higher instances of distorted RSSI signals. The results presented in Figure 10c were derived from the data of the second experiment collected by the tester holding an HTC M8 in Table 3. All of 126 steps were taken on the 54-m sampling route by the tester, only 116 were detected.

**Figure 11:**
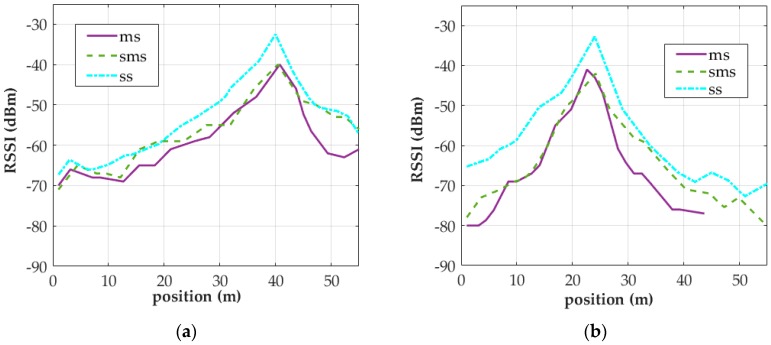
RSSI distributions of signals collected at the 5-GHz band by using different APs (the distributions were derived through the NVA algorithm). (**a**) AP1; (**b**) AP2.

Because RSSI distributions are subject to the accuracy of step detection algorithms, accuracy should be enhanced when RSSI signals are collected through MS. Two approaches were proposed based on this concept. One approach involves using two smartphones, one held in the hand to collect RSSI signals and the other attached to the lower back to acquire step detection signals. Both smartphones are controlled simultaneously via Bluetooth. In the other approach, the tester holds a smartphone to collect signals and manually counts the number of steps taken during sampling. The count is then entered into the step detection algorithm and an additional algorithm is used to calibrate IC. Figure 11 presents the RSSI distribution of AP signals collected at the 5-GHz band by a smartphone held in the hand to illustrate that the NVA perform well both at 2.4- and 5-GHz band. In this test, the accuracy of the step detection algorithm for detecting instances of IC was measured to be 100%. During both tests on the algorithm, over 100 AP signals were captured at the 2.4- and 5-GHz bands. This paper presents the RSSI distributions of only a few signals.

An analysis of the works discussed in Section 2 and a previous study [12] revealed that AP RSSI distributions improve the accuracy of indoor positioning algorithms to a higher degree than does RSSI value. In this study, the results from sampling experiments and the NVA algorithm suggest that the RSSI distributions of APs obtained through MS and SMS were similar to those obtained through SS. For online-phase positioning algorithms and the accuracy of online-phase positioning, a related discussion can be found in [12]. The present study demonstrated primarily the use of MS and SMS on an Android smartphone. RSSI fingerprints can be created during the construction of an indoor positioning system by using MS or SMS rather than SS, which involves more time and labor for system construction and maintenance. MS involves the least time for constructing and maintaining indoor positioning systems but requires familiarity with the sampling procedure to reduce human errors arising from the process, which could generate errors in the fingerprint database. SMS is more time-consuming than MS, but requires considerably less time and labor than SS and is less prone to errors.

## 5. Conclusions

This paper began with an introduction of technical details regarding the use of Android smartphones to collect AP beacon signals of Wi-Fi networks and common errors in the collection process and proceeded to propose and explicate a fast procedure for capturing Wi-Fi fingerprints. Three fingerprint collection method are discussed, namely SS, MS, and SMS. SS is a traditional method for radio fingerprint collection that involves considerable time and labor, which are primary factors in restricting its utilization in indoor positioning systems. In this study, AP signals were collected using SS, MS, and SMS. After the signals had been calibrated using the NVA algorithm, the RSSI distribution patterns of the signals became similar. Therefore, positioning algorithms that use MS or SMS to collect AP signals require less time for system building than do those that use SS for this purpose. In our signal-collecting experiment performed along a hallway 54 m in length, SS took over 2 h for signal collection, compared with MS and SMS, which took approximately 150 and 300 s, respectively. Based on these results, using SS to conduct AP signal collection in an entire building incurs prohibitive costs, whereas if MS or SMS is applied, AP signal collection becomes feasible. This study provides researchers who focus on the use of Android smartphones to conduct indoor positioning with an understanding of how to use simpler methods to collect signals and enhance the validity of research findings accordingly.

## Figures and Tables

**Table 1:** Mean received signal strength indicator (RSSI) values and standard deviations for three smartphones used to collect Wi-Fi signals from 4 access points (APs) for 60 s.

Device	AP1		AP2		AP3		AP4	
	Mean (dBm)	* S.D.	Mean (dBm)	S.D.	Mean (dBm)	S.D.	Mean (dBm)	S.D.
HTC M8	−38.15	1.65	−48.95	2.01	−62.8	3.47	−75.35	2.08
Samsung S5	−39.19	2.30	−43.63	1.65	−67.6	3.38	−69.85	1.79
Sony Z5	−30.69	2.66	−43.31	2.34	−65.81	1.18	−68.07	2.29

* S.D.: standard deviation (dBm).

**Table 2:** Amounts of time required to complete different sampling methods on a sampling route 54 m in length; SS: static sampling; MS: moving sampling; SMS: stepped MS.

Strategy	SS	MS	SMS
Time consuming	≈120 min.	≈150 sec.	≈300 sec.

**Table 3:** Test results of step-detection algorithm.

Strategies	HTC M8		Nokia 6	
	Test 1	Test 2	Test 1	Test 2
Hand	* 121/121	116/126	118/120	119/120
Lower back	118/118	127/127	119/119	121/121
Stabilizer	110/115	115/122	108/118	117/124

* Numbers of estimated/actual steps.
